# Distribution of Bacterial α1,3-Galactosyltransferase Genes in the Human Gut Microbiome

**DOI:** 10.3389/fimmu.2019.03000

**Published:** 2020-01-13

**Authors:** Emmanuel Montassier, Gabriel A. Al-Ghalith, Camille Mathé, Quentin Le Bastard, Venceslas Douillard, Abel Garnier, Rémi Guimon, Bastien Raimondeau, Yann Touchefeu, Emilie Duchalais, Nicolas Vince, Sophie Limou, Pierre-Antoine Gourraud, David A. Laplaud, Arnaud B. Nicot, Jean-Paul Soulillou, Laureline Berthelot

**Affiliations:** ^1^Microbiota Hosts Antibiotics and bacterial Resistances (MiHAR), Université de Nantes, Nantes, France; ^2^Laboratoire EA3826 Thérapeutiques cliniques et expérimentales des infections IRS2 Nantes Biotech, Université de Nantes, Nantes, France; ^3^Department of Emergency Medicine, CHU de Nantes, Nantes, France; ^4^Bioinformatics and Computational Biology, University of Minnesota, Minneapolis, MN, United States; ^5^Centre de Recherche en Transplantation et Immunologie UMR 1064, INSERM, Université de Nantes, Nantes, France; ^6^Institut de Transplantation Urologie Néphrologie (ITUN), CHU de Nantes, Nantes, France; ^7^CHU de Nantes, CIC 1413, Pôle Hospitalo-Universitaire 11 Santé Publique, Clinique des données, Nantes, France; ^8^Institut des Maladies de l'Appareil Digestif, CHU Nantes, Nantes, France; ^9^INSERM U1235, Nantes, France; ^10^Neurology department, CIC Neurology, CHU de Nantes, Nantes, France

**Keywords:** intestinal microbiome, α1,3-Galactosyltransferase, α1,3-Gal antigen, shotgun data, enterobacteriaceae

## Abstract

Because of a loss-of-function mutation in the GGTA1 gene, humans are unable to synthetize α1,3-Galactose (Gal) decorated glycans and develop high levels of circulating anti-α1,3-Galactose antibodies (anti-Gal Abs). Anti-Gal Abs have been identified as a major obstacle of organ xenotransplantation and play a role in several host-pathogen relationships including potential susceptibility to infection. Anti-Gal Abs are supposed to stem from immunization against the gut microbiota, an assumption derived from the observation that some pathogens display α1,3-Gal and that antibiotic treatment decreases the level of anti-Gal. However, there is little information to date concerning the microorganisms producing α1,3-Gal in the human gut microbiome. Here, available α1,3-Galactosyltransferase (GT) gene sequences from gut bacteria were selectively quantified for the first time in the gut microbiome shotgun sequences of 163 adult individuals from three published population-based metagenomics analyses. We showed that most of the gut microbiome of adult individuals contained a small set of bacteria bearing α1,3-GT genes. These bacteria belong mainly to the Enterobacteriaceae family, including *Escherichia coli*, but also to Pasteurellaceae genera, *Haemophilus influenza* and *Lactobacillus* species. α1,3-Gal antigens and α1,3-GT activity were detected in healthy stools of individuals exhibiting α1,3-GT bacterial gene sequences in their shotgun data.

## Introduction

Human beings and old-world primates are unable to express the α1,3-Galactose epitope because of a loss-of-function mutation in the GGTA1 gene (Glycoprotein α1,3-Galactosyltransferase 1) coding α1,3GalactosylTransferase [see (1) for review]. As such, humans develop anti-α1,3-Galactose (anti-Gal) antibodies (Abs) supposed to result from an immune response to gut microbiota within the first year of life (2, 3).

Humans have high levels of circulating Abs directed against the α1,3-Gal epitope, with more than 1% of B-cells exhibiting a membrane receptor devoted to α1,3-Gal recognition (4). Anti-Gal Abs have been identified in the 1980s as the main barrier to vascularized organ xeno-transplantation, where they act as the major etiological component of hyper-acute vascular xenograft rejection (5), which does not occur if the transplant comes from GGTA1 KO pigs (6). Anti-Gal Abs in humans have also been proposed as protective against a variety of Gal-positive microorganisms (7, 8) or viruses budding from non-human host cells (9) and likely contribute to several host-pathogen relationships, also including potential infection susceptibility. Anti-Gal may also play a role in some auto-immune processes. Patients with multiple sclerosis, with lower circulating titers of anti-Gal Abs (10) and with dysbiosis of the gut microbiota, may lack bacteria able to synthetize the α1,3-Gal epitope in their microbiota (11).

Importantly, presence of the α1,3-Gal epitope has been detected in engineered animal devices such as porcine or bovine biological heart valves (BHV) (12), and anti-Gal Abs may play a role in late valve deterioration, a major limitation of the success of BHV implantation in young adults. Thus, the level and type of microbiota able to synthetize the α1,3-Gal epitope may represent a key environmental factor controlling several pathological processes. The knowledge of the micro-organisms producing the α1,3-Gal antigens eliciting and maintaining blood anti-Gal levels may allow for the design of strategies to accordingly modulate antibody titers in some pathological states.

It is known that several bacteria are able to synthetize α1,3-Gal sugars (13) such as *Klebsiella pneumonia* (14), *Escherichia coli* (15), *Salmonella* (16), or *Serratia marcescens* (17) and that antibiotics can decrease the levels of blood anti-Gal in an experimental model (18). Yet, there is little information on which α1,3-Gal positive bacteria in the human gut microbiome initiate the primary response against the α1,3-Gal epitope in the first year of life, or which bacteria likely sustain the high level of anti-Gal antibodies in adults (19). In this paper, we analyzed for the first time the spectrum of α1,3GalactosylTransferase (α1,3GT) gene sequences in bacteria from human gut microbiome samples of 163 healthy adults using shotgun metagenomic analysis.

## Materials and Methods

### Metagenomic Shotgun Sequences

We meta-analyzed two published population-based metagenomics analyses to assess the presence of α1,3GalactosylTransferase (α1,3GT) sequences in the gut microbiomes of human subjects. First, we analyzed metagenomic shotgun sequences from the Human Microbiome Project (HMP), including 239 adult subjects (20). Raw data are available at http://hmpdacc.org/. Stool sequences (*n* = 106 individuals) of this first cohort were randomly selected using the sample function in R programming language, and ultimately submitted to the meta-analysis. Full metadata and annotation protocols are available on the HMP DACC website (http://hmpdacc.org/HMMCP). We used the raw sequences downloaded from the website.

We also analyzed data of a second and more recent shotgun-sequencing project from the LifeLines-DEEP cohort. The raw sequence data from this Dutch population-based cohort are available from the European genome-phenome archive (https://www.ebi.ac.uk/ega) at accession number EGAS00001001704. We randomly selected 57 individuals in the database and analyzed raw sequences downloaded from the EGA. We then analyzed a shotgun metagenomic DNA sequencing dataset of 20 samples from Hmong in Thailand (*n* = 15), Karen in Thailand (*n* = 5) from a multi-generational Asian American cohort (21).

### α1,3-GalactosylTransferase Gene Sequences

The gene sequences encoding α1,3-GalactosylTransferase (α1,3GT) enzymes were collected from the Bioinformatics Resource Portal ExPASy (https://enzyme.expasy.org/EC/2.4.1.87). We also collected all gene sequences corresponding to α1,3GT among the 4,800 described genomes of bacteria in the Kyoto Encyclopedia of Genes and Genomes (KEGG) (https://www.genome.jp/kegg/) and the gene sequences coding α1,3GT proteins in UniProt (https://www.uniprot.org/). Evolution has provided a high diversity of enzymes able to create the branched α1,3 position in galactose, which can stimulate an immune response in humans. Supplementary Table 1 (also commented in the result section) provides the final list of sequences submitted for bioinformatics analysis.

### α1,3GalactosylTransferase Phylogenic Tree Building

We retrieved 193 bacterial gene sequences of the α1,3GT from the KEGG and UniProt databases by using α1,3GT-related keywords and manually curated the obtained list (Supplementary Table 1). We found 55 duplicates of nucleotide sequences, 45 of which coming from different strains of *E. Coli*; the 10 remaining sequences were duplicates from the same genus except one, *Acidaminococcus intestinis* and *Campylobacter lari*. We removed the duplicates to conduct the alignment and the tree building as advised for these algorithms; the alignment was performed using Clustal Omega from the EMBL-EBI website (22, 23). Results in the FASTA format were then given as input for the tree building. We chose RaxML (v. 8.2.12) (24) to compute phylogenetic trees. It relies on Maximum Likelihood and bootstrapping to generate multiple trees and rank them from the most to the least likely. It allows for fast and reliable tree building, adapted to calculation on a cluster. Standard GTRCAT and rapid bootstrap analysis and search for best-scoring ML tree were the options provided to the algorithm. The RaxML output trees were handled and visualized with R (v. 3.5.1) with the help of the ggtree package (v. 1.14.6) (25, 26). Coloration was done with the order group rather than genus or family in order to highlight the similarities between species and facilitate the interpretation. Orders of the bacteria were retrieved using BacDive (27), a database containing diverse phenotypic information.

### Identification in Shotgun Sequences of α1,3-GalactosylTransferase Gene Sequences

All of available α1,3GT gene sequences from bacteria were used to quantify their abundance in the shotgun sequences from the fecal samples from the publicly available population-based metagenomics analyses of the two cohorts. A minimal sequence identity cutoff of 85 or 93.5% between the reference α1,3GT sequences and the shotgun reads sequences from the HMP and lifelines-DEEP datasets were both used in our assignment procedure. A 90%-confidence lowest common ancestor taxonomy assignment strategy was used to call taxonomy for each read whenever such a read mapped to the α1,3GT genes of multiple reference organisms.

### Bioinformatic Analysis

We processed and trimmed the raw sequences from the two metagenomic datasets for quality using SHI7 (28). We then used the BURST DNA aligner (https://github.com/knights-lab/BURST) in CAPITALIST mode at 85 and 93.5% identity against the representative set (i.e., α1,3GT bacteria gene sequences). BURST is an optimal, high-speed pairwise sequence aligner specialized in aligning many short reads against reference databases under a range of sequence similarity assumptions (28). The reason BURST was chosen is because it does not use any type of heuristic approximation in its alignment calculations, unlike other popular short read aligners designed for high-similarity matches only. Instead, it performs exhaustive pairwise alignment on all sequences using dynamic programming without statistical approximations, and hence avoids the need for ensemble analysis that may be required when using other approximate tools that may have sensitivity/specificity tradeoffs in pursuing or calculating alignments. Especially for lower alignment identities, general-purpose techniques designed for speedy approximate alignment (read mappers, classifiers) are not applicable, as they use approximations that are reasonable for short-read mapping but not lower-identity alignment (under some metrics of precision/accuracy/sensitivity/recall) (29–31). Because we are looking for potentially divergent sequences, we selected BURST over high-speed metagenomic classifiers such as MEGAN, MEGAN-LR (31) or Kraken (29) despite the increased computational cost. Here, our reference database was the collection of α1,3GT gene sequences collected from the literature, ExPASy, KEGG, and UniProt as described above.

### Detection of α1,3-Gal Antigen

For flow cytometry, cells were labeled with IB4 lectin coupled with FITC (Sigma Aldrich) at dilution 1:50 in PBS-BSA 1% at 4°C for 20 min. After washing, cells were analyzed using Celesta flow cytometer (BD Biosciences) and Flow Jo software.

Stool samples from 6 healthy volunteers were used. A written consent was obtained for all individuals and the study was approved by the local ethical committee. For ELISA, maxisorp plates (Nunc, Thermo Fisher Scientific) were coated with proteins at 20 μg/ml from the different stool lysates in PBS 2 h at 37°C. After washing with PBS, Tween 0.05%, blocking was performed using PBS-BSA 1% for 1 h. An IgM anti-α1,3-Gal (clone M86, Enzo) was incubated at dilution 1:50 in PBS-BSA 1% for 1 h at 37°C. For the revelation, an anti-IgM coupled with horse radish peroxidase 1/500 was added. The substrate TMB (BD biosciences) was then incubated and coloration was stopped with sulfuric acid (0.18 M, Sigma Aldrich). Plates were read at 450 nm using a Spark 10 M microplate reader (Tecan, Manndorf, Switzerland).

### α1,3-GT Activity

Maxisorp plates were coated with asialofetuin (the Galactose acceptor, Sigma Aldrich) at 20 μg/ml in carbonate buffer, 0.1 M pH 9.5. Blocking was performed using carbonate buffer, BSA 1%. Samples were incubated in 50 mM Tris pH 7.5, 10 mM CaCl_2_, 5 mM UDP-Gal, for 3 h at 37°C providing the α1,3-Gal precursor. The presence of added α1,3-Gal was revealed by addition of IgM anti- α1,3-Gal (clone M86, Enzo) was added.

### Statistical Analyses

Linear regression was assessed for correlation between the number of α1,3-GT gene sequences in the microbiome or the number of α1,3-GT gene positive bacteria and the amount of detected Gal antigen in stools of six healthy donors.

## Results

### Analysis of Genes Coding α1,3-GalactosylTransferases in Bacteria

Only few bacteria, mostly pathogens, were described expressing α1,3-GalactosylTransferase (α1,3GT) in the literature (13–17). This enzyme belongs to the glycosyltransferase family 8 according to the Carbohydrate Active enZymes (CAZy) classification (http://www.CAZy.org). Using the ExPASy and KEGG public databases, we were able to identify 193 bacteria species and subspecies harboring genes coding α1,3GT enzymes. Various short names have been attributed to these genes (WaaO, WaaI, WaaL, RfaI, RfaJ, gspA, or ybl158). Moreover, the corresponding α1,3GT enzymes bore also various functional descriptions such as “UDP-D-galactose:lipopolysaccharide-alpha-1,3-D-galactosyltransferase” or “General stress protein A,” requiring careful data collection (Supplementary Table 1). For these reasons, it is difficult to ensure exhaustive characterization of all genes encoding enzymes responsible for the production of the α1,3Gal antigen. Accordingly, our data provide a minimal estimation of the involved bacteria. When specified, the KEGG orthology numbers were KO3275 or KO3278, fitting with an α1,3GT function.

**Figure 1:**
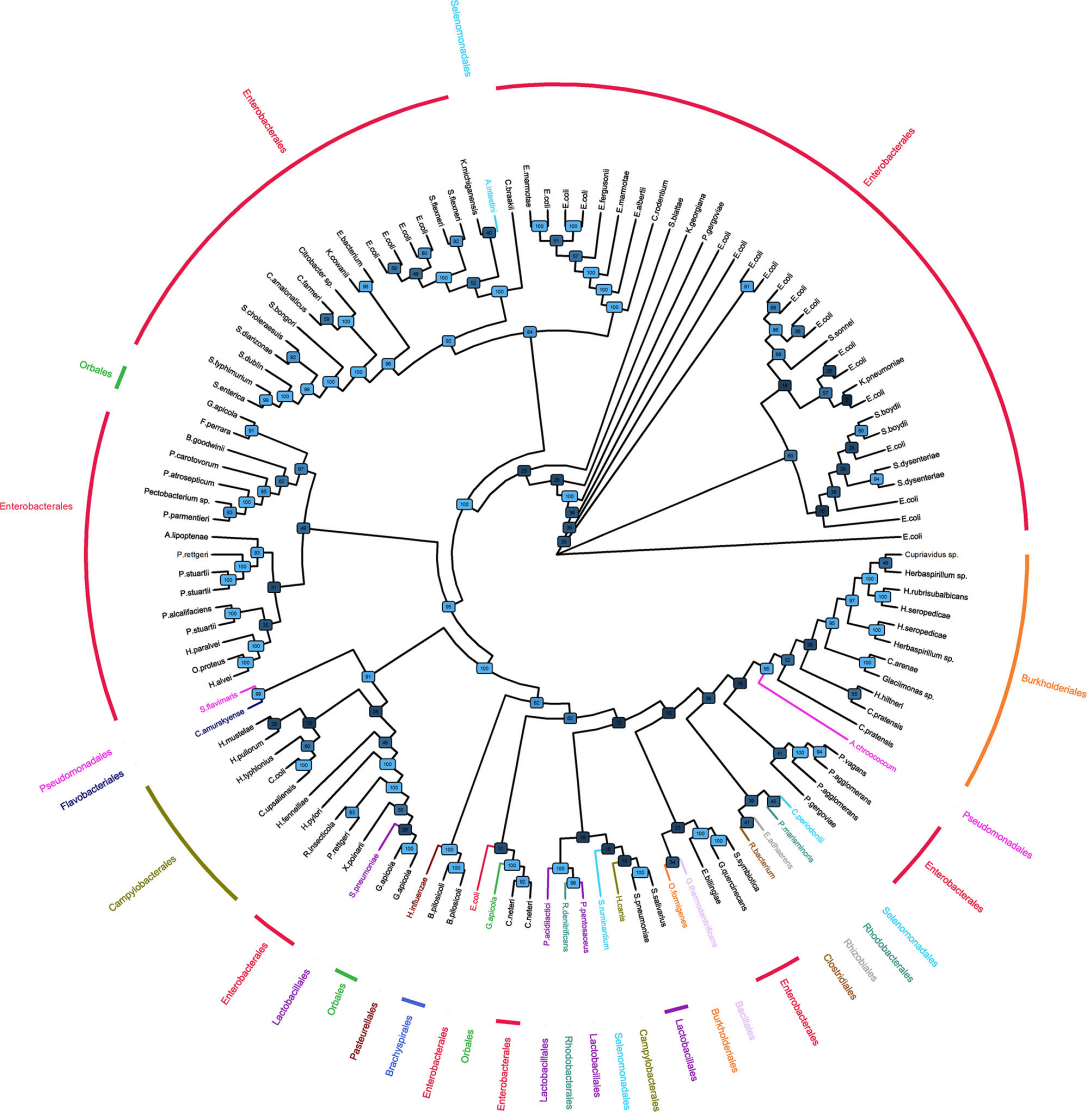
Phylogenic tree of bacteria bearing α1,3GT sequences. Phylogenetic tree of bacteria based on the α1,3-GalactosylTransferase sequences retrieved on KEGG and UniProt databases. Orders have been represented in different colors. The tree is represented as a cladogram; therefore, the interpretation of the groups is possible but the branch length has no meaning. Bootstrap confidence values are indicated on nodes.

The phylogenetic tree with alignment of bacterial α1,3GT sequences showed that sequences largely clustered with bacteria orders (Figure 1), indicating a classical transmission of α1,3GT genes. Enterobacterales (including *E. coli*) was the predominant order of bacteria bearing α1,3GT. As expected, genetic distance of bacterial α1,3GT to mammal α1,3GT sequences was important (data not shown, comparison with bovine, porcine, murine, canine, cat, monkey, sheep, rat, and human sequences of GGTA1 gene). Some bacteria appeared to bear at least 2 different α1,3GT gene sequences (for example *E. coli*) suggesting that several enzymes translated from different genes harbor α1,3GT function.

### Identification of α1,3GT Sequences in Gut Microbiome of Adult Individuals

Based on all α1,3GT bacteria sequences extracted from the ExPASy and KEGG databases, we then studied the presence of corresponding sequences in the gut microbiome using the open access shotgun data of the two different cohorts of adult individuals.

**Figure 2:**
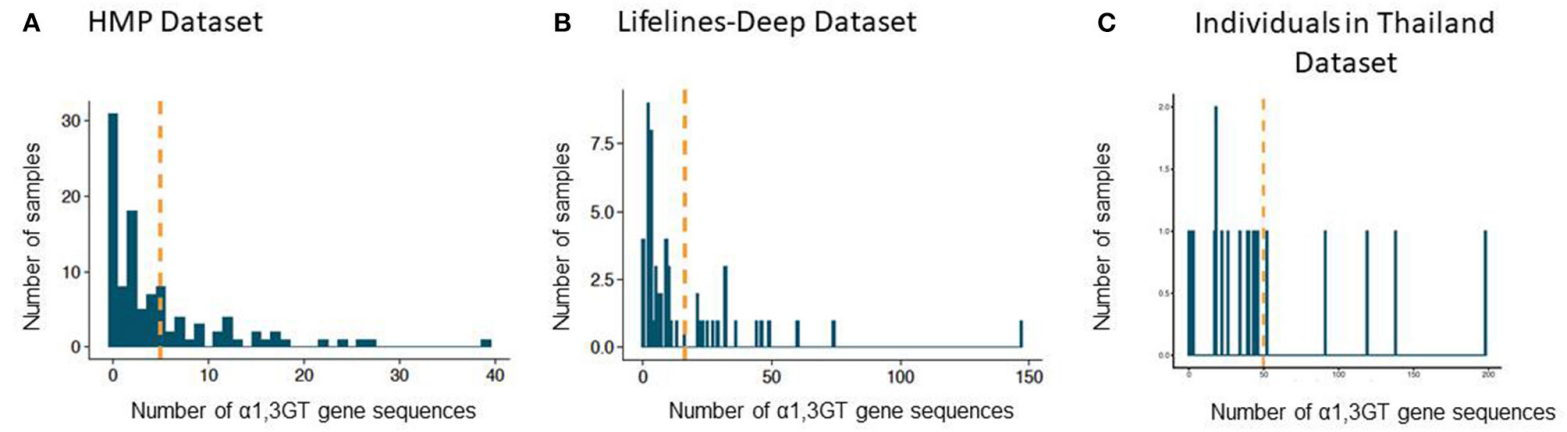
Presence of sequences with α1,3GT sequence homology in the gut microbiome of adult individuals from two cohorts. **(A)** Number of hits for α1,3GT sequences in the HMP dataset (106 individuals), mean number = 5. **(B)** Number of hits for α1,3GT sequences in DEEP lifelines dataset (57 individuals), mean number = 16. **(C)** Number of hits for α1,3GT sequences in Asian American dataset (20 individuals), mean number = 50. BURST was used with sequence identity homology cutoff of 0.85. The orange line represents the mean number of α1,3GT homologs present per patient.

In the Human Microbiome Project (HMP) cohort, 75 individuals (71% of the studied population) exhibited α1,3GT sequences in their gut microbiome when a lenient 0.85 sequence identity cutoff was used. The mean number of sequences above the homology threshold per subject was 5 (standard deviation: 7; range: 0–39) (Figure 2A). With a 0.935 sequence homology cutoff, the mean number sequences per subject α1,3GT was 2 (standard deviation: 4, range: 0–19).

In the more recent database from the LifeLines-DEEP cohort, we confirmed also the presence of α1,3GT sequences and found these sequences in 53 subjects (93%) with a 0.85 identity cutoff (Figure 2B). The mean number of sequences per subject was 16 (standard deviation: 24; range: 0–147). With a 0.935 cutoff, the mean number of compatible α1,3GT sequences was 11 (standard deviation: 19, range: 0–116).

In another recent database from a multi-generational Asian American cohort, where we only selected individuals living in Thailand (*n* = 20), we and found α1,3GT sequences in 19 subjects (95%) with a 0.85 identity cutoff (Figure 2C). The mean number of sequences per subject was 50 (standard deviation: 50; range: 0–147). With a 0.935 cutoff, the mean number of compatible α1,3GT sequences was 11 (standard deviation: 19, range: 0–198).

### α1,3GT Positive Bacteria in Gut Microbiome of Adult Individuals

The α1,3GT sequences allowed us to identify a first bacteria map of the human gut microbiome which exhibited α1,3GT genes. Using BURST, we found that optimal lowest common ancestor taxonomy assignment mostly associated the α1,3GT homology to sequences originating from the Enterobacteriaceae family, mostly *Escherichia coli*; the Pasteurellaceae family, mostly *Haemophilus influenza*; the *Streptococcus* genus, mostly *Streptococcus salivarius* and *Streptococcus pneumoniae*; and the Lactobacillaceae family, with most *Pediococcus* and *Lactobacillus* genera (Table 1). Most, but not all, bacteria species displaying the α1,3GT gene sequences were classified as Gram-negative. Interestingly, gram-negative bacteria bear a complex lipopolysaccharide (LPS) in the outer leaflet of their membrane, which can harbor α1,3Gal antigen.

**Table 1:** Most frequent hits for taxonomy.

**Taxonomy (BURST based lowest common ancestor taxonomy assignment on the basis of a 85% of homology with the α1,3GT sequence)**	**Number and ranking of homologous α1,3GT sequences**
k__Bacteria;p__Proteobacteria;c__Gammaproteobacteria;o__Enterobacterales;f__Enterobacteriaceae;g__Escherichia;s__Escherichia coli;t__	2141
k__Bacteria;p__Proteobacteria;c__Gammaproteobacteria;o__Enterobacterales;f__Enterobacteriaceae	1792
k__Bacteria;p__Proteobacteria;c__Gammaproteobacteria;o__Pasteurellales;f__Pasteurellaceae;g__Haemophilus;s__Haemophilus influenzae;t__	251
k__Bacteria;p__Proteobacteria;c__Gammaproteobacteria;o__Enterobacterales;f__Enterobacteriaceae;g__Escherichia	147
k__Bacteria;p__Proteobacteria;c__Gammaproteobacteria;o__Enterobacterales;f__Enterobacteriaceae;g__Escherichia;s__Escherichia fergusonii;t__Escherichia fergusonii ATCC 35469	130
k__Bacteria;p__Firmicutes;c__Bacilli;o__Lactobacillales;f__Streptococcaceae;g__Streptococcus;s__Streptococcus salivarius	81
k__Bacteria;p__Proteobacteria;c__Gammaproteobacteria;o__Enterobacterales;f__Hafniaceae;g__Hafnia;s__Hafnia alvei;t__Hafnia alvei ATCC 13337	68
k__Bacteria;p__Firmicutes;c__Bacilli;o__Lactobacillales;f__Lactobacillaceae;g__Pediococcus;s__Pediococcus_pentosaceus	64
k__Bacteria;p__Firmicutes;c__Bacilli;o__Lactobacillales;f__Streptococcaceae;g__Streptococcus;s__Streptococcus_pneumoniae	61
k__Bacteria;p__Bacteroidetes;c__Bacteroidia;o__Bacteroidales;f__Tannerellaceae;g__Parabacteroides;s__Parabacteroides distasonis;t__	68
k__Bacteria;p__Bacteroidetes;c__Bacteroidia;o__Bacteroidales;f__Tannerellaceae;g__Parabacteroides	64
k__Bacteria;p__Proteobacteria;c__Gammaproteobacteria;o__Enterobacterales;f__Enterobacteriaceae;g__Escherichia;s__Escherichia coli	52
k__Bacteria;p__Firmicutes;c__Bacilli;o__Lactobacillales;f__Lactobacillaceae;g__Lactobacillus;s__Lactobacillus delbrueckii;t__Lactobacillus delbrueckii subsp. bulgaricus	32
k__Bacteria;p__Proteobacteria;c__Gammaproteobacteria;o__Enterobacterales;f__Hafniaceae;g__Hafnia	32
k__Bacteria;p__Bacteroidetes;c__Bacteroidia;o__Bacteroidales;f__Prevotellaceae;g__Prevotella;s__[Hallella] seregens;t__Hallella seregens ATCC 51272	31
k__Bacteria;p__Bacteroidetes;c__Bacteroidia;o__Bacteroidales;f__Prevotellaceae;g__Prevotella;s__Prevotella sp. P6B1;t__	30
k__Bacteria;p__Proteobacteria;c__Gammaproteobacteria;o__Enterobacterales;f__Hafniaceae;g__Hafnia;s__Hafnia alvei	23
k__Bacteria;p__Firmicutes;c__Clostridia;o__Clostridiales;f__Eubacteriaceae;g__Eubacterium;s__[Eubacterium] eligens;t__[Eubacterium] eligens ATCC 27750	21
k__Bacteria;p__Bacteroidetes;c__Bacteroidia;o__Bacteroidales;f__Prevotellaceae;g__Prevotella	19

*A lowest common ancestor assignment and an α1,3GT sequence identity cutoff of 85% in the HMP, DEEP-lifelines, Asian American datasets were used for the analysis*.

### α1,3GT Positive Gut Microbiome of Healthy Individuals Exhibit α1,3GT Activity and α1,3Gal Antigen Production

**Figure 3:**
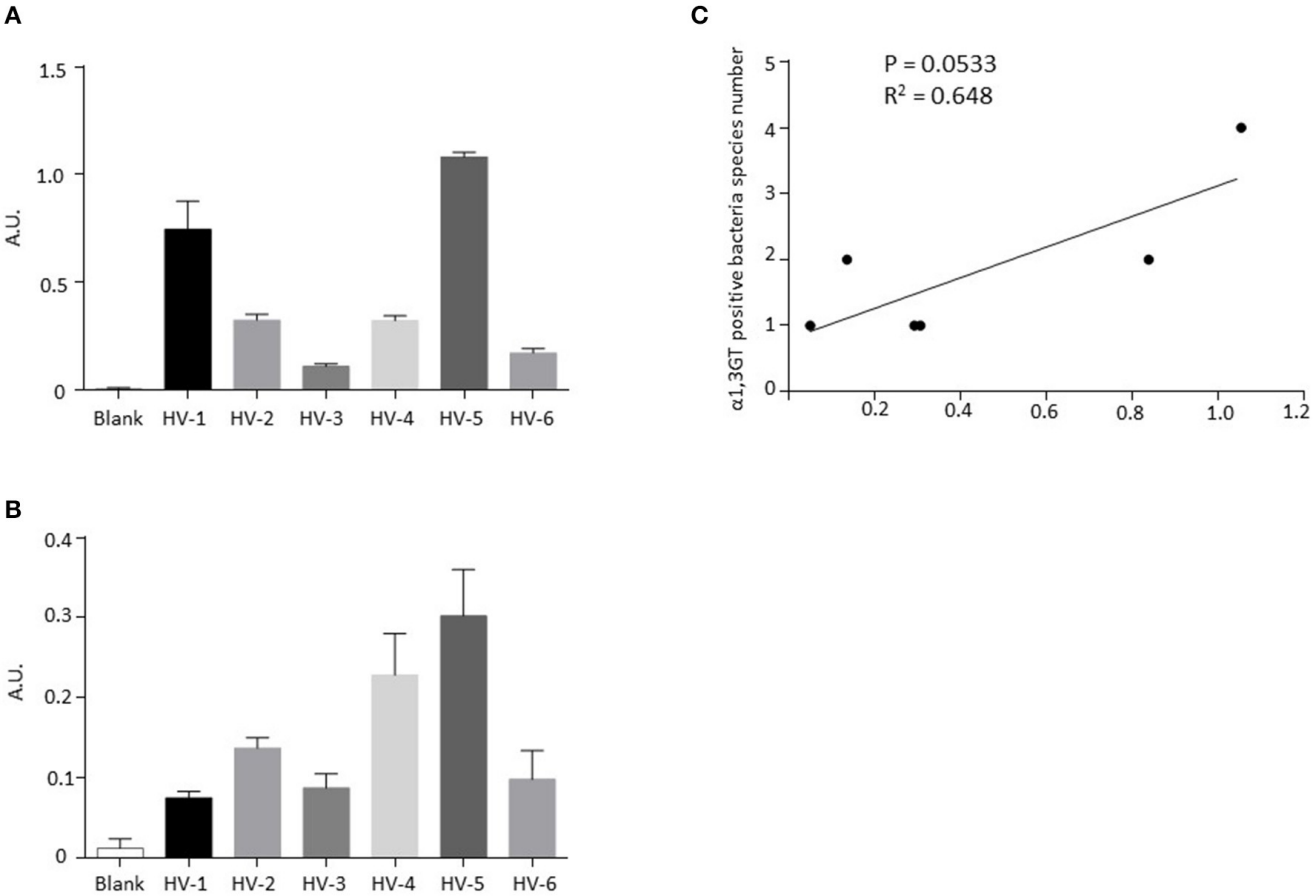
Detection of α1,3-Gal antigen and α1,3GT activity in stools from healthy donors exhibiting bacterial α1,3GT gene sequences in their shotgun analysis. **(A)** Levels of α1,3-Gal antigen detected by ELISA in stools from 6 healthy volunteers. **(B)** Activity of α1,3GT enzymes detected by ELISA in stools from six healthy volunteers. **(C)** Correlation between the number of detected bacteria species harboring α1,3GT gene sequences and the amounts of Gal antigen in stools.

To confirm our bioinformatics sequence analysis on gut microbiome from healthy individuals, we tested by ELISA the presence of α1,3Gal antigen on human stool samples. First, a validation was performed on human cells (peripheral blood mononuclear cells) and rat splenocytes, used, respectively, as negative and positive controls. As shown in Supplementary Figure 1A, IB4 lectin, specific for α1,3Gal antigen, bound to rat cells and not to human cells and the presence of α1,3Gal antigen by ELISA was only detected in rat cells (Supplementary Figure 1B). Stool samples from six healthy volunteers (HV), all characterized as positive for bacterial α1,3GT gene sequences (Table 2), exhibited α1,3Gal antigen production (Figure 3A) and α1,3GT activity (Figure 3B). There was no significant correlation between the number of hits for α1,3GT sequences in shotgun data and the amount of Gal antigen in stools in this small cohort (data not shown). However, there was a tendency for positive correlation (*P* = 0.0533, *R*^2^ = 0.648, Figure 3C) between the number of bacteria species harboring α1,3GT sequences and the amount of Gal antigen in the stools detected by ELISA, suggesting that the extend of diversity of bacteria with α1,3GT in the microbiota, influences the production of Gal antigen.

**Table 2:** Characteristics of healthy volunteers.

	**HV-1**	**HV-2**	**HV-3**	**HV-4**	**HV-5**	**HV-6**
Age (Years)	51	53	51	67	70	73
Sex (Male or Female)	F	F	F	F	F	F
Presence of α1,3GT sequences in shotgun data	+	+	+	+	+	+
Hits for α1,3GT sequences in shotgun data (Number)	4	1	125	38	60	11
Detected bacteria species with α1,3GT sequences (Number)	2	1	1	1	4	2

## Discussion

Here, we describe for the first time the distribution of α1,3GT gene homologs in the human gut microbiome expected to encode the Gal antigen using a meta-analysis of two publicly available shotgun data sets including 163 adults from United States and The Netherlands. This method, contrary to 16S RNA analysis of gut microbiota which we previously used (11), directly detects the presence of α1,3GT gene sequences instead of relying on taxonomic prediction (29). We showed that sequences with an 85 or 93.5% homology with the reference α1,3GT gene sequences which encode the α1,3-Gal antigen were restricted to a small number of gut microbiota, dominated by the Enterobacteriaceae family. High homology with the α1,3GT gene sequences was also identified in the *Haemophilus* and *Streptococcus* genera, which represent <1% of the human gut microbiome in relative abundance (30). Altogether, our approach suggests that only a small fraction of the gut microbiome of adult individuals is able to produce the Gal antigen. Our study fits with previous observations that indirectly suggested that gut microbiome was the main source of production of the Gal antigen (18) resulting in the rise of the anti-Gal within the first year of life. Interestingly, in α1,3GT-KO mice, modifications of gut microbiome followed during the first months of life were related to anti-Gal Ab levels (31).

However, our estimation likely underestimates the actual proportion of the microbiota able to produce the Gal antigen as also suggested by the presence of anti-Gal antibodies in most of the adult individuals tested. Indeed, it is possible that a fraction of adult individuals in whom no high homology of known α1,3GT sequences were identified (29 and 7% in the two cohorts, respectively) corresponds to “false negative,” owing the high diversity of the sequences of α1,3GT in bacteria and not characterized viruses/parasites in the microbiome. Moreover, not all genes coding α1,3GT have been identified in bacteria as such. For instance, in *Neisseria meningitidis*, anti-Gal antibodies bind to pili from these bacteria (32) but the corresponding enzyme with α1,3GT activity is not described. Besides, enzymes previously thought to be unimodal are now being annotated with multiple modalities. Indeed, two genes coding other glycosyltransferases were recently described as coding bispecific transferases exhibiting α1,3GT activity: wciN in *Streptococcus pneumoniae* (33) and WclR in Escherichia coli (34) (Supplementary Table 1).

Although anti-Gal Abs are found in most individuals, there is a substantial dispersion of their levels attributed to several demographic variables, including ABO blood groups, as well as age, sex, geographic location during childhood, and diet (35, 36). Another important variable may be the exposure to different types and levels of microorganisms of the healthy microbiota. Several bacterial pathogens able to synthetize α1,3-Gal such as *Escherichia coli* (34), *Streptococcus pneumonia* (33) *Salmonella* (16) or parasites may also contribute to immunization in the childhood or adulthood in specific pathological contexts. Moreover, inflammatory bowel diseases and gastrointestinal infections are particularly associated with adherent-invasive *Escherichia coli* (37–39). Oral inoculation of α1,3GT gene KO mice with this bacterium induces production of anti-Gal Abs (40). Moreover, this bacterium readily binds human anti-Gal Abs (13). Altogether, our data suggest that α1,3GT positive bacteria of the gut microbiota, generate anti-Gal antibodies previously described to involve a substantial fraction of the B cell repertoire (4) and may also shape the dispersion of blood anti-Gal antibody levels. However, our present study only portraits a first map of the bacteria of gut microbiota harboring α1,3GT sequences allowing the synthesis of α1,3Gal epitope. Beside the nature of the bacteria themselves, which may influence the immunogenicity of the α1,3GT in the gut, other parameters, particularly the direct assessment of the actual amounts of α1,3GT antigen in the gut microbiota, would be important to correlate with the levels of blood anti-Neu5Gc in the future. Unfortunately, such study would require large cohorts of individuals with both blood and gut microbiota samples. We could only provide here preliminary results based on a few cases suggesting that the level of diversity of the bacteria harboring α1,3GT gene sequences have a borderline correlation with the levels of α1,3Gal in the stools.

We have recently suggested that the low level of anti-Gal reported in multiple sclerosis (10) could be linked to the low level of α1,3GT positive flora also observed in their gut microbiome (11). It is possible that manipulation of anti-Gal antibodies can find some utility in human autoimmune diseases and particularly in multiple sclerosis, a disease of unknown precise etiology. The present study is thus a step toward a possible demonstration that these decreased levels were governed by abnormal microbiota. One can hypothesize that supplementing multiple sclerosis patient diet with Enterobacteriaceae or other α1,3GT positive species could be considered in the future. However, currently, one cannot speculate on a direct role of either the anti-Gal antibodies or rather that anti-Gal could be a marker of a microbiota alteration directly interfering in this disease (41). Furthermore, the link between the anti-Gal antibody levels and the amount of bacterial displaying α1,3GT activity is currently speculative.

Taken together, our data may open new avenues for delineating targeted strategies to eventually modulate the level of anti- α1,3Gal antibody response in some diseases (10, 11), attenuate food allergy to α1,3Gal antigen, or decrease the risk of deleterious effects of anti-α1,3Gal Abs after implantation of animal derived bio-devices (42) such as bovine or porcine bio-prosthetic heart valves or help fighting some infections due to Gal displaying microorganisms. Manipulating the microbiome to decrease the level of anti- α1,3Gal Abs may be also useful for xenotransplantation, as using genetically engineered GGTA1 gene KO animal donors in the clinic is not yet permitted.

## Conclusions

This study described for the first time the gut bacteria species bearing α1,3-GT gene sequences: the Enterobacteriaceae family, including *Escherichia coli*, Pasteurellaceae genera, *Haemophilus influenza*, and *Lactobacillus* species. These bacteria were detected in the gut microbiome of most individuals. Moreover, this bioinformatics analysis was confirmed by biological tests on human stool samples. α1,3-Gal antigens and α1,3-Galactosyltransferase activity were detected in healthy stools of individuals exhibiting α1,3-Galactosyltransferase bacterial sequences in their shotgun data. These data could offer more target strategies to modulate anti-Gal antibodies in patients confronted to animal derived biotherapeutics or biodevices and could represent an important issue for xenotransplantation and auto-immune diseases in the future.

## Data Availability Statement

All datasets generated for this study are included in the article/Supplementary Material.

## Author Contributions

EM was in charge of shotgun data analysis and alignment of sequences. EM, GA-G, and QL performed bioinformatics analysis of shotgun data. CM performed ELISA and flow cytometry experiments. YT and ED collected fecal samples in healthy individuals. BR performed the research of α1,3-GT sequences. VD, AG, and RG performed phylogenic tree building. LB, NV, and SL made a substantial contribution to data management and analysis. P-AG, DL, and AN were involved in revising the manuscript critically and made a substantial contribution to the final manuscript. LB and J-PS designed all the experimental work, coordinated the study. EM, J-PS, and LB wrote the manuscript.

### Conflict of Interest

The authors declare that the research was conducted in the absence of any commercial or financial relationships that could be construed as a potential conflict of interest.

## Supplementary Material

Supplementary Figure 1 Confirmation of α1,3-Gal antigen presence in rat cells and not in human cells. **(A)** Detection of α1,3-Gal antigen on cell surface using IB4 lectin coupled with FITC by flow cytometry. **(B)** Level of α1,3-Gal antigen detected by ELISA on cell lysates. ^*^*p* < 0.05, Kruskal-Wallis test.

Supplementary Table 1 List of bacteria harboring α1,3GT genes. When available, the gene name and the KEGG orthology numbers are indicated.
