# Supplementary figures and images for: Distribution of Bacterial α1,3-Galactosyltransferase Genes in the Human Gut Microbiome
Source: Front Immunol. 2020 Jan 13;10:3000. doi: 10.3389/fimmu.2019.03000 (PMC6970434; doi:10.3389/fimmu.2019.03000)

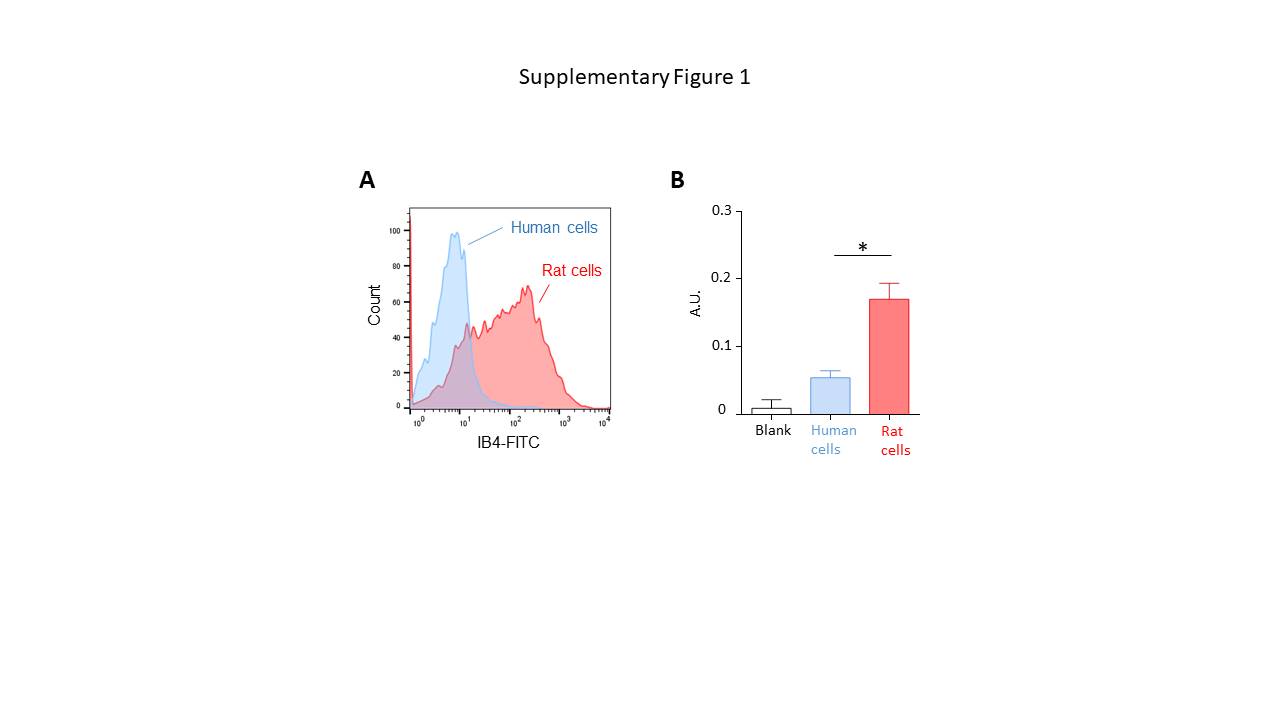

Supplement: Supplementary Figure 1 — Confirmation of α1,3-Gal antigen presence in rat cells and not in human cells. (A) Detection of α1,3-Gal antigen on cell surface using IB4 lectin coupled with FITC by flow cytometry. (B) Level of α1,3-Gal antigen detected by ELISA on cell lysates. *p < 0.05, Kruskal-Wallis test. [file Image_1.jpeg]
